# Change in Interpersonal and Metacognitive Skills During Treatment With Cognitive Behavioral Analysis System of Psychotherapy and Metacognitive Therapy: Results From an Observational Study

**DOI:** 10.3389/fpsyt.2021.619674

**Published:** 2021-08-16

**Authors:** Svenja Sürig, Katharina Ohm, Ulrike Grave, Sarah Glanert, Philipp Herzog, Eva Fassbinder, Stefan Borgwardt, Jan Philipp Klein

**Affiliations:** ^1^Department of Psychiatry, Psychosomatics and Psychotherapy, University of Lübeck, Lübeck, Germany; ^2^Department of Psychiatry and Psychotherapy, Christian-Albrechts-University Kiel, Kiel, Germany; ^3^Department of Psychology, University of Greifswald, Greifswald, Germany

**Keywords:** depression, interpersonal skills, social cognition, metacognitive beliefs, metacognitive therapy, cognitive behavioral analysis system of psychotherapy

## Abstract

**Background:** Interpersonal skills deficits and dysfunctional metacognitive beliefs have been implicated in the etiology and maintenance of depression. This study aimed to investigate the association between changes in these skills deficits and change in depressive symptoms over the course of treatment with Cognitive Behavioral Analysis System of Psychotherapy (CBASP) and Metacognitive Therapy (MCT).

**Methods:** In this prospective, parallel group observational study, data was collected at baseline and after 8 weeks of an intensive day clinic psychotherapy program. Based on a shared decision between patients and clinicians, patients received either CBASP or MCT. Ninety patients were included in the analyses (CBASP: age *M* = 38.7, 40.5% female, MCT: age *M* = 44.7, 43.3% female). Interpersonal deficits were assessed with the short-form of the Luebeck Questionnaire for Recording Preoperational Thinking (LQPT-SF) and the Impact Message Inventory (IMI-R). Metacognitive beliefs were assessed with the Metacognition Questionnaire-30 (MCQ-30). The Quick Inventory of Depressive Symptomatology (QIDS-SR16) was utilized to assess depressive symptoms. A regression analysis was conducted to assess variables associated with outcome. ANCOVAs were utilized to investigate whether improvement in skills deficits is dependent on type of treatment received.

**Results:** Improvements in preoperational thinking and increases in friendly-dominant behavior were associated with change in depressive symptoms. There was no association between reductions in dysfunctional metacognitive beliefs and a decrease in depressive symptoms. While both treatment groups showed significant improvements in interpersonal and metacognitive skills, there was no significant between-group difference in the change scores for either of these skills.

**Conclusion:** Our findings suggest that changes in interpersonal skills seem to be of particular relevance in the treatment of depression. These results have to be replicated in a randomized-controlled design before firm conclusions can be drawn.

## Introduction

Depression is one of the most common psychiatric disorders (1) and associated with a great burden of disease (2). Its course is often recurrent (3), treatment-resistant (4) and about one third of patients with depression experience a chronic course (5). According to DSM-5, persistent depressive disorder (PDD) can be diagnosed when symptoms are present for at least 2 years and symptom-free intervals have never lasted more than 8 weeks at a time (6).

Numerous factors that contribute to the etiology and maintenance of depression have been suggested (7), e.g., dysfunctional expectations (8, 9). Moreover, these factors include skills deficits targeted by “third wave” behavioral therapies that are associated with depression but not captured by the existing diagnostic criteria. Skills deficits that are targeted in these modern psychotherapies include deficits in interpersonal skills (theoretically intended to be primarily targeted in Cognitive Behavioral Analysis System of Psychotherapy (CBASP; 10)) and metacognitive skills (theoretically intended to be primarily targeted in Metacognitive Therapy (MCT; 11)). A better understanding of the associations of changes in these skills deficits with outcome might contribute to improving existing psychotherapies for depression (10).

CBASP was specifically developed as a treatment for PDD (11, 12) and is recommended as a first line treatment by the European Psychiatric Association (13). Numerous studies demonstrate the effectiveness of CBASP in the treatment of PDD (14, 15). With an emphasis on utilizing the relationship between patient and therapist as a therapeutic tool, CBASP addresses the interpersonal deficits of chronically depressed individuals (11, 12). Patients with depression have been found to exhibit hostile and submissive interpersonal behavior and these behavior patterns are even more pronounced in individuals suffering from PDD (16). This hostile-submissive behavior might be associated with experiences of early emotional abuse (17) and this association might be mediated by a specific deficit in social cognition termed preoperational thinking (17). The term preoperational thinking was originally coined by Piaget's theory of cognitive-affective development (18). McCullough proposes that chronically depressed individuals exhibit a perceptual and behavioral disconnection from their environment as they are unable to perceive the consequences of their interpersonal behavior and adapt it accordingly. Being entrapped in the present moment and unable to disengage from an egocentric worldview makes it impossible to effectively connect with others (11). This global and prelogical way of thinking has been summarized in the words of one chronically depressed patient: “Whatever I do, nothing will ever change” (19). Utilizing the Luebeck Questionnaire for Recording Preoperational Thinking (LQPT) as a measure specifically developed to assess preoperational thinking (20), studies found higher levels of preoperational thinking in chronically depressed patients compared to episodically depressed patients and healthy controls (17, 19–22). Further, preoperational thinking is associated with early emotional abuse and this association might be mediated by interpersonal fears (19).

MCT is a transdiagnostic treatment approach based on the assumption that perseverative thinking styles underlie psychopathology (23). According to the metacognitive model of depression, depressive symptomatology is maintained by inflexible and maladaptive thinking styles, called the cognitive attentional syndrome (CAS). The CAS comprises rumination and worry, threat monitoring and dysfunctional coping behaviors and is maintained by positive (i.e., concerning the usefulness of engaging in the CAS) and negative (i.e., concerning the uncontrollability and danger of thoughts) metacognitive beliefs (23, 24). Studies could show that negative metacognitive beliefs contribute to certain symptoms of depression (i.e., rumination) (25, 26). Depressive rumination however maintains and exacerbates depressed mood (27, 28). Also, negative metacognitive beliefs have been found to prospectively predict depression (29). MCT aims to identify and modify negative repetitive thinking as well as dysfunctional metacognitive beliefs (30). Meta-analyses have found MCT to be effective in the treatment of depression (31, 32).

In summary, there is convincing evidence for the contribution of interpersonal and metacognitive skills deficits to depression and for the efficacy of CBASP and MCT in treating depression. Fewer studies evaluated these skills deficits as underlying treatment mechanisms. Patients treated with CBASP have been found to exhibit more friendly-dominant behaviors after treatment (33, 34). Studies investigating MCT as a treatment for depression could show that negative metacognitive beliefs decreased over the course of treatment (35, 36). There are few studies that investigated the association between changes in skills deficits and changes in depressive symptomatology. Decreases in hostile-submissive behaviors have been found to be associated with depression reduction (37, 38). Constantino et al. (39) tested a mediation model and could show that higher therapeutic alliance predicted decreases in hostile-submissiveness which in turn related to less depressive symptomatology in patients treated with CBASP. Examining the efficacy of MCT and implicated change mechanisms, Hjemdal et al. (40) could show that change in dysfunctional metacognitive beliefs predicted change in depression from pre-treatment to 1-year follow-up. To our knowledge, thus far no study examined whether change in preoperational thinking predicts reduction in depressive symptoms. Furthermore, there are no studies comparing the relative contribution of changes in interpersonal skills and changes in metacognitive skills to outcome.

Therefore, this present study aims to investigate the associations between interpersonal skills deficits as well as dysfunctional metacognitive beliefs and depressive symptomatology. We hypothesize that improvement in interpersonal as well as metacognitive skills is associated with a decrease in depressive symptoms. Further, we aim to investigate whether changes in interpersonal and metacognitive skills are specific to the respective type of treatment. We hypothesize that patients treated with CBASP show a greater improvement in interpersonal skills compared to patients treated with MCT while patients treated with MCT show a greater improvement in metacognitive skills. Another aim of this study was to develop and validate a short-form of the LQPT to assess preoperational thinking that allows for more time-efficient administration and facilitates administration for depressed individuals that may struggle with diminished abilities to concentrate.

## Materials and Methods

### Patient Sample

This prospective, parallel group observational study uses data from the ICARE study (Investigating Care Dependency And its Relation to OutcomE) that aims to investigate the German version of the Care Dependency Questionnaire (41). The ICARE study was conducted in accordance with the Declaration of Helsinki and approved by the ethics committee of the University of Lübeck (reference number 17-049). Patients were recruited from the day treatment program for depression at the Department of Psychiatry and Psychotherapy, University of Lübeck, Germany. The treatment program focuses on treating depressive disorders with CBASP or MCT and lasts for 8 weeks. Patients did not receive financial compensation.

Inclusion criteria were the presence of a depressive disorder diagnosis as well as a minimum age of 18 and adequate understanding of the German language. Exclusion criteria were acute suicidality at admission, a known diagnosis of an organic mental disorder, schizophrenia, schizotypal disorder or delusional disorder, bipolar disorder, primary diagnosis of substance abuse or substance dependence of alcohol, cannabinoids, sedatives, cocaine, or hallucinogens, or a physical illness requiring immediate treatment. As we aimed to only include patients who were not yet familiar with the treatment program, patients were excluded if they had been admitted to the day clinic in the previous 12 months. Informed written consent was obtained from all patients. The diagnosis of depressive disorders was done by utilizing a diagnostic interview that was based on DSM-5 (42).

**Figure 1:**
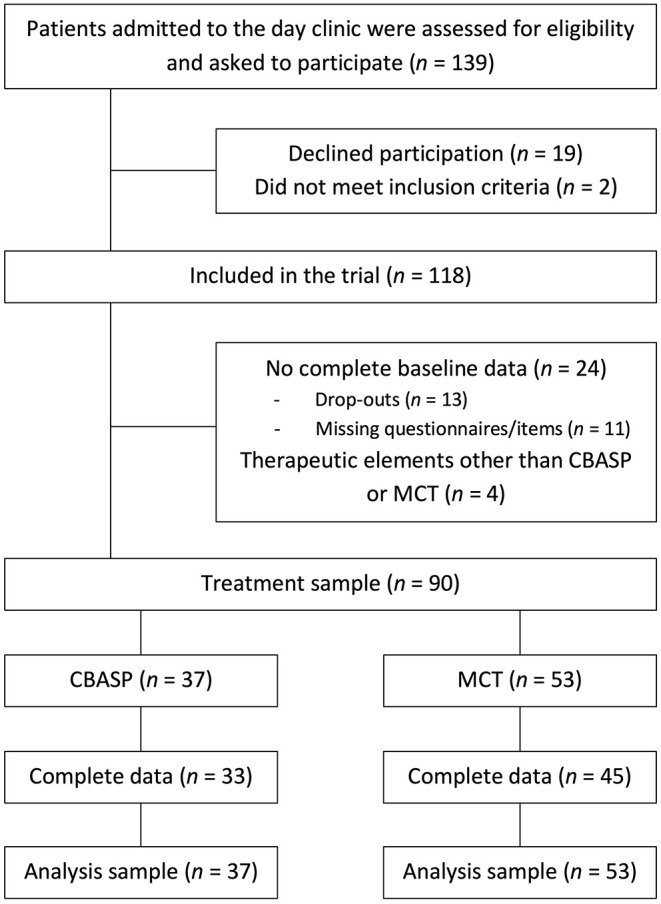
Patient flow.

Recruitment began in January 2019 and ended in January 2020. A full patient flow can be found in Figure 1. Briefly, 139 patients were assessed for eligibility and asked to participate and 90 patients were included in the analyses.

### Intervention

Patients received 8 weeks of intensive treatment with either CBASP or MCT. The decision between CBASP and MCT was made in a shared decision-making process between the clinician and the patient after the intake interview, based on the following algorithm: diagnosis of the patient (e.g., patients with a PDD were recommended to choose CBASP while patients with comorbid diagnosis of anxiety or obsessive-compulsive disorder were recommended to choose MCT), presenting complaint (e.g., patients with primary interpersonal difficulties received a recommendation for CBASP while patients with primary worry and rumination received a recommendation for MCT) and the patient's preference. Patients received specific therapeutic elements unique to the treatment modality (CBASP or MCT) including one session of individual therapy with a psychologist or medical doctor, one session of group therapy with a psychologist or medical doctor and one session with a nurse specialist per week. Therapy is delivered under weekly team supervision and a weekly visit by a senior physician. In addition, all patients received further multimodal interventions, including physical therapy, occupational therapy as well as a nurse specialist group focusing on mindfulness training. They also received guideline-adherent pharmacotherapy (43).

#### Cognitive Behavioral Analysis System of Psychotherapy (CBASP)

CBASP aims to teach patients to reduce preoperational thinking and engage in more adaptive interpersonal behaviors (11). Situation analyses constitute a central therapeutic element alongside with disciplined personal involvement of the therapist. In an operationalized procedure patient and therapist revisit interpersonal situations with the aim of challenging preoperational thinking and developing new behavioral alternatives. Interpersonal discrimination exercises are aimed at increasing safety in the therapeutic relationship by demonstrating the differences between the therapist's responses to certain patient behaviors and the responses of maltreating significant others. A prototypical treatment with CBASP comprised the following therapeutic elements: list of significant others, transference hypothesis, situation analyses and disciplined personal involvement of the therapist.

#### Metacognitive Therapy (MCT)

MCT is a transdiagnostic treatment approach based on the assumption that perseverative thinking styles constitute a maintaining factor for several psychiatric disorders. A disorder specific case formulation and treatment procedure for depression was developed (30). MCT focuses on identifying and modifying negative repetitive thinking styles such as worry and rumination. For the case formulation, trigger thoughts, worry and rumination, threat monitoring strategies and maladaptive coping behaviors are explored alongside with their maintaining positive and negative metacognitive beliefs. Attentional Training Technique (ATT) as well as Detached Mindfulness (DM) constitute central elements of MCT that aim at modifying the control of attention and heightening metacognitive awareness of inner experiences and the detachment from maladaptive thoughts and beliefs. A prototypical treatment with MCT comprised the following therapeutic elements: metacognitive case formulation, DM, ATT and worry/rumination postponement.

### Instruments

#### Luebeck Questionnaire for Recording Preoperational Thinking Short-Form (LQPT-SF)

The LQPT is a self-assessment instrument developed to record preoperational thinking as a specific cognitive psychopathology of individuals suffering from PDD. In its original version, it consists of 20 items each in the form of written scenarios depicting difficult interpersonal situations. Participants are required to choose between two response options reflecting either a high or a low level of preoperational thinking (e.g., “Nobody likes me. I am always disappointed by others. I cannot rely on others.” indicating a preoperational response to a friend canceling a dinner invitation as opposed to “Too bad my friend cannot come. I hope he is well. I will call him tomorrow and ask what is going on.” or “I knew he never liked me, this only proves it.” as a preoperational response to not being invited to a birthday party as opposed to “I will call my neighbor. I would like to go to this party.”). Items are scored 0 and 1 with a low total score indicating a high level of preoperational thinking. In several studies, the LQPT has been shown to be a reliable and valid instrument (Cronbach's α = 0.90) (20, 21, 44). The LQPT is publically available online (https://bit.ly/3q3zqGd).

As completing the LQPT can be time-consuming as well as demanding for participants due to its length, we aimed to devise a short-form of the LQPT (LQPT-SF). A principal component analysis for categorical variables (CAT-PCA) performed on the LQPT scores of sample data gathered by Klein et al. (19) resulted in the retention of one component with an eigenvalue of 7.86 that was able to explain 39.30 % of variance. Eleven items with very good or better loadings on this component were summarized in the LQPT-SF, namely item 2, 5, 6, 7, 9, 11, 12, 14, 16, 18, and 21 (45). The LQPT-SF demonstrated excellent internal consistency (Cronbach's α = 0.91) as well as acceptable convergent construct validity as indicated by significant correlations with relevant IMI subscales of chronically depressed patients (submissive subscale, *r* = −0.44, hostile subscale, *r* = −0.37, friendly-dominant subscale, *r* = 0.50). Further, the LQPT-SF showed excellent discriminant abilities as evidenced by significant results for all comparisons: PDD (M = 4.91, SD = 3.34) vs. ED (M = 8.00, SD = 3.02), *t* = −4.03, *p* < 0.001, PDD vs. HC (M = 10.60, SD = 0.72), *t* = −11.04, *p* < 0.001, and ED vs. HC, *t* = −4.51, *p* < 0.001, with effect sizes of 0.95, 2.12 and 1.18, respectively. For the effect sizes, Hedge's g was calculated for the pairwise comparisons due to differences in sample sizes (46). In this present study, the LQPT-SF was completed at baseline and end of treatment.

#### Metacognition Questionnaire-30 (MCQ-30)

The MCQ-30 is a self-report questionnaire that assesses metacognitive beliefs, judgements and monitoring tendencies (47). It consists of 30 items that are rated on a four-point Likert scale ranging from 1 (do not agree) to 4 (agree very much), e.g., “My worrying is dangerous for me.” There are five subscales (cognitive confidence, positive beliefs about worry, cognitive self-consciousness, negative beliefs concerning uncontrollability and danger, need to control thoughts). The MCQ-30 demonstrates good internal consistency (Cronbach's α = 0.91) as well as good validity (47). Patients completed the MCQ-30 at baseline and end of treatment.

#### Impact Message Inventory (IMI-R)

The IMI-R is a transactional instrument used to assess interpersonal impact messages according to the dimensions of the interpersonal circumplex model (48). Following the assumption of interpersonal complementarity, treating clinicians completed the IMI to assess the participants' interpersonal behavior patterns at baseline and end of treatment. The IMI-R has 56 items that are rated on a four-point Likert scale ranging from 1 (not at all) to 4 (very much so). Items begin with the phrase “When I am with this person, he/she makes me feel…,” followed by e.g., “…distant from him/her” as a sample hostile item or “…in charge” as a sample submissive item (49). The IMI-R demonstrates good psychometric properties as Cronbach's α coefficients for the octants range from 0.68 to 0.86 (50). Following previous research, we focus on the hostile-submissive and friendly-dominant subscale of the IMI-R (37, 39).

#### Quick Inventory of Depressive Symptomatology (QIDS-SR16)

The QIDS-SR16 is a self-assessment instrument that contains 16 items assessing depressive symptomatology of the last seven days. Items are scored from 0 to 3 with higher scores reflecting greater impairment. For three domains (sleep, appetite/weight and restlessness/agitation) only the highest scored item is included in the total score. Total QIDS-SR16 scores range from 0 to 27 with total scores indicating the following: scores of 5 or lower no depression, 6 to 10 mild depression, 11 to 15 moderate depression, 16 to 20 severe depression and scores greater 21 very severe depression (51). The German version of the QIDS-SR16 demonstrates adequate internal consistency (Cronbach's α = 0.77) (52). Patients completed the QIDS-SR16 on a weekly basis.

#### Childhood Trauma Questionnaire Short-Form (CTQ-SF)

The CTQ-SF is a self-report instrument that assesses childhood maltreatment before the age of 18 (53). It contains 28 items that can be summarized in five subscales of emotional abuse, physical abuse, sexual abuse, emotional neglect and physical neglect. Items are rated on a 5-point Likert scale ranging from never true to very often true (e.g., “When I was growing up people in my family said hurtful or insulting things to me”). The German version of the CTQ-SF demonstrated good internal consistency as evidenced by Cronbach's α = 0.94 (54). The CTQ-SF was completed at baseline.

### Statistical Analyses

Statistical analyses were conducted using SPSS 24.0 (55). If not otherwise specified, statistical tests were evaluated as two-sided tests with significance levels set at *p* < 0.05. A modified intention-to-treat analysis was employed using all participants with complete baseline data. Individual missing values were replaced with the individual participant mean for the respective scale if the number of missing items was <20% (56). Missing sum scores were replaced using the mean of the posterior distribution from the fully conditional specification method obtained by iterative Markov Chain Monte Carlo estimation (57) using 20 imputations per missing value. Prior to conducting the analyses, relevant assumptions were tested.

Paired sample *t*-tests were conducted to investigate pre-post treatment differences in depressive symptomatology (assessed by the QIDS), interpersonal skills (assessed by the LQPT-SF and the IMI-R) and metacognitive skills (assessed by the MCQ-30).

We conducted a hierarchical multiple regression analysis in order to investigate variables significantly associated with change in depressive symptomatology as assessed by change in QIDS scores over the course of therapy. In a first step, baseline scores of the QIDS and of all variables assessing skills deficits were entered in order to control for their influence. In a second step, change scores of the LQPT-SF, hostile-submissive and friendly-dominant IMI as well as MCQ-30 were entered in the model. We tested the assumptions of the regression analysis by examining independence of residuals, linearity, homoscedasticity, multicollinearity and normal distribution.

In order to assess whether improvement in interpersonal skills (assessed by the LQPT-SF and the IMI-R) and metacognitive skills (assessed by the MCQ-30) is dependent on type of treatment intervention (CBASP vs. MCT), several ANCOVAs were conducted with the relevant outcome measure (LQPT-SF, IMI-R and MCQ-30) as dependent variable and therapeutic concept (CBASP vs. MCT) as predictor while controlling for the respective baseline scores. We tested the assumptions of the ANCOVAs by examining linearity, homogeneity of regression slopes and variances, normal distribution and homoscedasticity. *Post-hoc* power analyses were conducted with G^*^Power 3.1 by calculating f^2^ and setting alpha at 0.05 (58).

We conducted sensitivity analyses for the hierarchical regression model as well as for the ANCOVAs to correct for potential confounding variables. All variables where we found baseline imbalances were included as covariates (age, onset of depression, comorbid disorder, exposure to childhood adversity). To correct for baseline differences regarding the presence of comorbid disorders, the presence of anxiety (ICD-10 F40 or F41 diagnosis) or obsessive-compulsive disorder (ICD-10 F42 diagnosis) was combined in one variable (comorbid diagnosis).

Effect size estimates for repeated measures were calculated by taking the correlation between pre- and post-scores into account. For the regression analysis, f^2^ was calculated to estimate the effect size of adding individual variables to the model by dividing the squared partial correlation of an individual variable by its reciprocal. Cohen's f^2^ will be interpreted as f^2^ = 0.02 indicating a small effect, f^2^ = 0.15 indicating a medium effect and f^2^ = 0.35 indicating a large effect (59).

## Results

### Sample Characteristics

Detailed clinical characteristics of the sample can be found in Table 1. Thirty-seven patients were treated with CBASP compared to 53 patients being treated with MCT. Seventy-three percent of all patients were diagnosed with PDD. Patients treated with CBASP more often suffered from early onset depression (<21 years) and were younger compared to patients treated with MCT. Also, they reported higher rates of emotional abuse as assessed by the CTQ-SF. Treatment groups did not differ significantly in gender, marital status, language, school education or employment status (Table 1).

**Table 1:** Demographic and clinical characteristics.

	**All**		**CBASP**		**MCT**		
**Variables**	**Mean**	**SD**	**Mean**	**SD**	**Mean**	**SD**	**Test statistic U**
Age in years	42.21	12.70	38.65	12.70	44.70	12.22	1246.50^*^
Number of previous episodes	6.94	8.04	7.17	6.51	6.78	9.02	649.00
**CTQ-SF**							
Total	46.89	16.24	50.20	17.00	44.80	15.56	520.50
Emotional abuse	11.70	5.80	13.77	6.21	10.46	5.22	467.50^*^
Physical abuse	7.23	3.61	7.81	3.82	6.83	3.44	527.50^+^
Sexual abuse	5.64	1.74	5.60	1.67	5.66	1.80	628.00
Emotional neglect	14.19	5.53	15.31	4.73	13.41	5.94	550.50
Physical neglect	8.80	3.54	9.47	4.20	8.33	2.95	625.00
	**N**	**%**	**N**	**%**	**N**	**%**	**Test statistic** **χ** ^2^ ^a^
Female gender	38	42.2	15	40.5	23	43.4	1.48
Unemployed	41	45.6	19	51.4	22	41.5	0.85
Chronic depression	66	73.3	30	81.1	36	67.9	1.93
Early onset of depression	50	55.6	29	78.4	21	39.6	13.25^*^
**Marital status**							6.29
Married	32	35.6	8	21.6	24	45.3	
Single	45	5.0	21	56.8	24	45.3	
Divorced	13	14.4	8	21.5	5	9.4	
**School education**							1.57
Lower	24	26.7	9	24.3	15	28.3	
Middle	32	35.5	13	35.1	19	35.9	
Higher	15	16.7	7	18.9	8	15.1	
Highest	18	20.0	8	21.6	10	18.9	
**Number of comorbid disorders**							
No comorbid disorder	16	18.2	6	16.2	11	21.2	2.15
1	44	50.5	19	41.4	25	48.1	
2	17	19.3	9	24.3	8	15.4	
3	11	12.5	3	8.1	8	15.4	
**Comorbid disorders**							
Substance use	8	8.9	3	8.1	5	9.4	0.05
Psychotic disorders	2	2.2	2	5.4	0	–	2.93
Anxiety disorders	23	25.6	5	13.5	18	34.0	4.79^*^
OCD	7	7.8	0	–	7	13.2	5.30*^a^
Trauma	10	11.1	2	5.4	8	15.1	2.07
Somatoform disorders	2	2.2	1	2.7	1	1.9	0.07
Eating disorders	1	1.1	1	2.7	0	–	1.45
Personality disorders	11	12.2	7	18.9	4	7.5	2.63

*CBASP, cognitive behavioral analysis of psychotherapy; MCT, metacognitive therapy; OCD, obsessive compulsive disorder; CTQ-SF, childhood trauma questionnaire short-form. Test statistics were computed to compare CBASP with MCT. ^+^p < 0.10*,

* *p < 0.05*.

a *Fisher's exact p-value was investigated if cell counts were <5*.

### Statistical Analyses

#### Pre-post Differences

At the end of treatment, patients exhibited significantly less depressive symptomatology as assessed by the QIDS compared to their admission, *t*_(52)_ = 6.51, *p* < 0.01, *d* = −0.69 (95% CI [−0.99 to −0.38]). They also exhibited less preoperational thinking, *t*_(52)_ = −6.53, *p* < 0.001, *d* = 0.69 (95% CI [0.38–0.98]), increases in friendly-dominant behaviors, *t*_(52)_ = −4.38, *p* < 0.001, *d* = 0.46 (95% CI [0.15–0.74]), and lower levels of dysfunctional metacognitive beliefs, *t*_(52)_ = 7.56, *p* < 0.001, *d* = −0.80 (95% CI [−1.06 to −0.45]). There was no significant change in hostile-submissive behaviors, *t*_(52)_ = 1.00, *p* = 0.32, *d* = −0.10 (95% CI [−0.39 to −0.19]). Effect size measures indicate a large pre-post effect of change in depressive symptoms for CBASP patients, *t*_(52)_ = 5.33, *p* < 0.001, *d* = −0.88 (95% CI [−1.27 to −0.65]), and a medium effect for patients treated with MCT, *t*_(52)_ = 4.13, *p* < 0.001, *d* = −0.57 (95% CI [−0.84 to −0.24]). On a descriptive level, patients treated with CBASP seem to show a larger decrease in preoperational thinking compared to MCT patients while patients treated with MCT exhibited a larger decrease in dysfunctional metacognitive beliefs (Table 2). Concerning the subscales of the MCQ-30, both patient groups demonstrate the largest improvement in the negative metacognitive beliefs subscale (MCT: M = 4.19, SD = 3.68, CBASP: M = 1.99, SD = 3.49) followed by improvements in the need to control thoughts subscale (MCT: M = 2.78, SD = 3.87, CBASP: 1.82, SD = 3.12).

**Table 2:** Outcome variables.

	**All**				**CBASP**				**MCT**			
**Variables**	**Mean**	**SD**	***t***	***d***	**Mean**	**SD**	***t***	***d***	**Mean**	**SD**	***t***	***d***
**QIDS**												
Week 0	14.52	5.54			14.62	5.23			14.45	5.80		
Week 8	10.78	5.59			10.12	6.13			11.24	5.20		
Difference	3.74	5.45	6.51^*^	−0.69	4.50	5.13	5.33^*^	−0.88	3.21	5.66	4.13^*^	−0.57
**LQPT-SF**												
Week 0	6.28	2.99			6.10	2.87			6.40	3.10		
Week 8	7.98	2.91			8.27	2.92			7.78	2.91		
Difference	1.70	2.47	−6.53^*^	0.69	2.17	2.56	−5.15^*^	0.85	1.39	2.39	−4.21^*^	0.58
**MCQ-30**												
Week 0	73.06	14.56			69.55	12.64			75.52	15.41		
Week 8	63.72	12.68			62.85	12.44			64.33	12.93		
Difference	9.34	11.71	7.56^*^	−0.80	6.69	8.83	4.61^*^	−0.76	11.19	13.13	6.21^*^	−0.86
**IMI Hos-Sub**												
Week 0	2.58	0.45			2.59	0.48			2.58	0.44		
Week 8	2.54	0.49			2.57	0.58			2.52	0.43		
Difference	−0.04	0.41	1.00	−0.10	−0.03	0.43	0.35	−0.05	−0.06	0.41	1.02	−0.15
**IMI Fri-Dom**												
Week 0	2.35	0.51			2.31	0.48			2.37	0.54		
Week 8	2.57	0.49			2.57	0.53			2.58	0.46		
Difference	0.22	0.48	−4.38^*^	0.46	0.25	0.46	−3.35^*^	0.57	0.20	0.50	−2.92^*^	0.42

*CBASP, cognitive behavioral analysis of psychotherapy; MCT, metacognitive therapy; QIDS, quick inventory of depressive symptomatology; LQPT-SF, luebeck questionnaire for recording preoperational thinking short-form; MCQ-30, metacognition questionnaire 30; IMI, impact message inventory completed by therapists; Hos-Sub, hostile submissive subscale; Fri-Dom, friendly dominant subscale. Effect sizes d were calculated for pre-post differences*.

* *p < 0.001*.

#### Variables Associated With Outcome

##### Main Analysis

The full model of the multiple regression analysis was statistically significant, *R*^2^ = 0.51, *F*_(9, 80)_ = 9.24, *p* < 0.001, adjusted *R*^2^ = 0.46. Adding the change variables to the model led to a statistically significant increase in *R*^2^ = 0.23, *F*_(4, 80)_ = 9.29, *p* < 0.001. The LQPT-SF change scores, B =0.73, SE = 0.22, *p* = 0.001, as well as friendly-dominant IMI change scores, B = 4.06, SE = 1.29, *p* = 0.002, were significantly associated with QIDS change. For each change of one unit in LQPT-SF change scores, the average mean in the change of QIDS change is about 0.73 with all other variables held constant. For each change of one unit in friendly-dominant change scores, the average mean in the change of QIDS change is about 4.06 with all other variables held constant. Baseline QIDS scores, B = 0.55, SE = 0.10, *p* < 0.001, and baseline friendly-dominant IMI scores, B = 3.28, SE = 1.35, *p* = 0.02, were significant contributors to the model. Improvement in metacognitive skills as assessed by the MCQ-30 was not significantly associated with QIDS change, *p* = 0.26. Rerunning the analysis with MCQ-30 negative metacognitive beliefs about the uncontrollability of rumination subscale instead of MCQ-30 total score yielded essentially the same results (B = 1.74, SE = 0.18, *p* = 0.33). For the main analysis, we also calculated f^2^ as a measure of effect size for the individual independent variables. LQPT-SF change scores and friendly-dominant change scores yielded effect sizes of f^2^ = 0.14 and f^2^ = 0.12, respectively, indicating a medium effect (Table 3).

**Table 3:** Regression analysis.

	**Model 1**				**Model 2**				
**Variables**	***B***	***SE***	**95 % CI**	***t***	***B***	***SE***	**95 % CI**	***t***	***f^**2**^***
QIDS W0	0.55	0.11	[0.33, 0.76]	4.97^**^	0.55	0.10	[0.35, 0.74]	5.50^**^	0.38
LQPT-SF W0	−0.03	0.20	[−0.42, 0.36]	−0.15	0.26	0.20	[−0.14, 0.66]	1.30	0.02
MCQ-30 W0	−0.05	0.04	[−0.13 to 0.04]	−1.04	−0.04	0.05	[−0.14, 0.05]	−0.91	0.01
IMI Hos-Sub W0	−0.67	1.43	[−3.51, 2.17]	−0.47	0.22	1.43	[−2.63, 3.06]	0.15	<0.001
IMI Fri-Dom W0	1.78	1.24	[−0.68, 4.25]	1.44	3.28	1.35	[0.59, 5.97]	2.43^*^	0.07
Δ LQPT-SF	–	–	–	–	0.73	0.22	[0.29, 1.16]	3.31^*^	0.14
Δ MCQ-30	–	–	–	–	0.06	0.05	[-0.04, 0.16]	1.14	0.02
Δ IMI Hos-Sub	–	–	–	–	−0.19	1.36	[−2.90, 2.53]	−0.14	<0.001
Δ IMI Fri-Dom	–	–	–	–	4.06	1.29	[1.50, 6.63]	3.15^*^	0.12
*R^2^ (adjusted)*	0.28 *(0.24)*				0.51 *(0.46)*				
ΔR*^2^*	0.28				0.23				
Δ*F*	6.60				9.29				

*QIDS, quick inventory of depressive symptomatology; LQPT-SF, luebeck questionnaire for recording preoperational thinking short-form; MCQ-30, metacognition questionnaire 30; IMI, impact message inventory completed by therapists; Hos-Sub, hostile submissive subscale; Fri-Dom, friendly dominant subscale*.

** *p < 0.001*,

* *p < 0.05*.

##### Sensitivity Analysis Investigating the Impact of Baseline Imbalances

When repeating the regression analysis and including variables with baseline imbalances to correct for potential confounding effects, neither age, B = = 0.03, SE = 0.04, 95% CI [−0.12, 0.05], *p* = 0.45, nor onset of depression, B = −1.26, SE = 1.17, 95% CI [−3.60, 1.08], *p* = 0.29, presence of comorbid diagnosis, B = −0.16, SE = 1.11, 95% CI [−2.37, 2.05], *p* = 0.88, nor emotional abuse, B = 0.01, SE = 0.10, 95% CI [−0.18, 0.21], *p* = 0.88, were significant predictors of QIDS change. Results for the remaining variables remained essentially the same (LQPT-SF change scores: B = 0.69, SE = 0.24, 95% CI [0.22–1.16], *p* = 0.004, friendly-dominant IMI change scores: B = 3.70, SE = 1.46, 95% CI [0.79–6.60], *p* = 0.01).

##### Sensitivity Analysis With Follow-Up Data

Using follow-up data, a sensitivity analysis was conducted by repeating the main analysis but utilizing the change in depressive symptoms from baseline to follow-up as the dependent variable. Thirty-six patients completed a 10-month follow-up (CBASP: *n* = 15, MCT: *n* = 21). The unstandardized effect size of friendly-dominant IMI change scores was similar to the one obtained in the main analysis, B = 3.66, SE = 1.34, 95% CI [−3.24, 10.56] but the variable did not reach significance (*p* = 0.29). LQPT-SF change scores were not significantly associated with QIDS change at follow-up, B = −0.05, SE = 0.60, CI [−1.28, 1.18], *p* = 0.94. Baseline QIDS scores remained a significant predictor of the model, B = 0.53, SE = 0.26, 95% CI [0, 1.05], *p* = 0.05.

#### CBASP vs. MCT

Contrary to expectations, treatment group did not significantly predict change in preoperational thinking, *F*_(1, 87)_ = 2.05, *p* = 0.16, η_p_^2^ = 0.02, hostile-submissive, *F*_(1, 87)_ = 0.19, *p* = 0.67, η_p_^2^ = 0.002, as well as friendly-dominant behaviors, *F*_(1, 87)_ = 0.06, *p* = 0.81, η_p_^2^ = 0.001, and was not a significant predictor of change in metacognitive skills, *F*_(1, 87)_ = 0.08, *p* = 0.37, η_p_^2^ = 0.009, when controlling for the respective baseline scores. Effect size measures as indicated by η_p_^2^ revealed a small effect of treatment group for LQPT-SF change scores, η_p_^2^ = 0.02. *Post-hoc* power analyses revealed low power to detect effects of 1 - β ranging from 0.06 to 0.30. Baseline scores of LQPT-SF, *F*_(1, 87)_ = 21.65, *p* < 0.001, η_p_^2^ = 0.20, hostile-submissive, *F*_(1, 87)_ = 13.42, *p* < 0.001, η_p_^2^ = 0.13, as well as friendly-dominant IMI scores, *F*_(1, 87)_ = 33.01, *p* < 0.001, η_p_^2^ = 0.28, and baseline MCQ-30 scores, *F*_(1, 87)_ = 34.80, *p* < 0.001, η_p_^2^ = 0.29, were each significant predictors of the respective change score. Following the intention-to-treat approach, all patients with baseline data were included in the analyses. Upon closer inspection of the data, there was one patient presenting with unusual values for the LQPT-SF change scores. Rerunning the ANCOVA and excluding this patient did not lead to a significant change in results but to an increase in *F*_(1, 86)_ = 3.00, *p* = 0.09, η_p_^2^ = 0.03.

##### Sensitivity Analysis Investigating the Impact of Baseline Imbalances

Treatment group was not a significant predictor of change in preoperational thinking, *F*_(1, 74)_ = 1.31, *p* = 0.26, η_p_^2^ = 0.02, hostile-submissive, *F*_(1, 74)_ = 0.04, *p* = 0.62, η_p_^2^ = 0.003, or friendly-dominant behavior, *F*_(1, 74)_ = 1.78, *p* = 0.19, η_p_^2^ = 0.02, and also did not significantly predict change in metacognitive skills, *F*_(1, 74)_ = 0.83, *p* = 0.37, η_p_^2^ = 0.01, when correcting for baseline imbalances.

## Discussion

### Summary of Results

In this study, we examined the association between change in interpersonal as well as metacognitive skills and depressive symptomatology during treatment with CBASP and MCT. Improvements in preoperational thinking as well as increases in friendly-dominant behaviors were associated with change in depressive symptoms. There was no association between change in dysfunctional metacognitive beliefs or hostile-submissive behavior and a reduction in depressive symptoms. Contrary to our expectations, treatment groups did not differ in the magnitude of change in interpersonal and metacognitive skills. The LQPT-SF appears to be a reliable and valid instrument as demonstrated by high internal consistency, convergent validity with relevant IMI subscales and excellent discriminant abilities.

### Comparison to Existing Studies

We were the first to demonstrate that improvements in preoperational thinking are associated with outcome as postulated by the CBASP model (11). Our present results extend the findings of Sondermann et al. (22), who also suggest the implication of preoperational thinking in depressive symptom severity as they found a high degree of preoperational thinking to be associated with a higher severity of depressive symptoms over an observation period of 2 years.

In line with previous research (33, 34, 39), patients treated with CBASP exhibited more friendly-dominant behaviors at the end of treatment. However, contrary to Constantino et al. (37, 39), increases in friendly-dominant and not decreases in hostile-submissive behaviors were associated with change in depressive symptoms. While several studies report decreases of hostile-submissive behaviors over the course of treatment with CBASP (33, 34, 39), patients in our sample did not exhibit significant changes in the hostile-submissive subscale. Possibly, differences in treatment duration may account for these conflicting results. The treatment program offered in this study entailed 8 weeks of treatment with a weekly individual and group session according to the therapeutic concept. However, CBASP as adapted for inpatient treatment usually involves 12 weeks of treatment with biweekly sessions (60) and other studies have followed this procedure (33, 34, 61). Increases in friendly-dominant behaviors that may be expressed in increased abilities to express one's own needs may be more readily detectable by others while decreases in hostile-submissive behaviors may need more time to manifest themselves. Thus, possibly we would have also detected change in hostile-submissive behaviors, if patients had been treated for a longer period of time in higher frequency, as this would have allowed more time for treatment effects to take place. This reasoning may also explain why Brakemeier et al. (34) could show stronger increases in friendly-dominant behaviors and greater reductions in depression severity over the course of treatment with CBASP. Nevertheless, improvements in interpersonal skills may contribute to changes in social relationships that have been found associated with reduced probability of relapse in patients treated with CBASP (62).

Patients did improve in dysfunctional metacognitive beliefs, but these decreases were not associated with decreases in depressive symptoms. Hjemdal et al. (40) and Faissner et al. (63) found that reductions in metacognitive beliefs predicted change in depression. However, Faissner et al. (63) also reported that changes in the Dysfunctional Attitudes Scale (DAS) were a better predictor of changes in depressive symptoms than changes in the MCQ-30 subscales. Considering that items of the DAS touch into interpersonal areas that are relevant in CBASP (e.g., “People will probably think less of me if I make a mistake”) these results may support our notion that especially changes in interpersonal skills seem to be of relevance for a reduction in depressive symptoms. The DAS has been found to exhibit significant high correlations with the LQPT (64).

Contrary to expectations, changes in skills deficits were not specific to the type of treatment received. This result can be interpreted against the background of the contextual model of psychotherapy (65). This model argues against the notion that certain specific ingredients are necessary for the success of a therapy. Rather, the contextual model argues that the the ingredients of therapy will be succesfull as long as the patient accepts their rationale and believes in their effectiveness. A competing explanation for our findings is that specific ingredients are indeed necessary for the success of therapy but treatment duration in our study was too short for these specific ingredients to achieve their full effect. On a different note, the group context of the clinical setting may offer experiences of interpersonal effective behavior irrespective of the treatment group. Not only does group psychotherapy foster interpersonal learning (66), but common spaces of the day clinic also promote engagement with others. Positive interpersonal interactions thus may contribute to improvement in interpersonal skills for patients treated with MCT. Also, improvements in dysfunctional metacognitive beliefs may influence changes in interpersonal behaviors and vice versa. According to the metacognitive model of depression, depressive rumination is associated with heightened perseverative self-focused attention (30). Thus, disengaging from ruminative thoughts and challenging dysfunctional metacognitive beliefs may open up attentional resources previously occupied with self-focused attention and enable active engagement with the social environment. ATT as utilized in the present treatment has been found effective in reducing self-focused attention (67). Patients treated with CBASP also exhibited significant improvements in dysfunctional metacognitive skills. As depressed patients are described to suffer from stressful interpersonal experiences which they at least in part generate themselves (68), improvements in interpersonal skills may lead to more positive interpersonal experiences. Assuming that there are less stressful interpersonal situations to ruminate about, dysfunctional metacognitive beliefs concerned with the uncontrollability of rumination may be likely to decrease. Also, a diffusion of skills taught in group and individual therapy may contribute to our results (e.g., CBASP patients might have learned about MCT skills from their fellow patients).

### Strengths and Limitations

The present study yields several strengths: First, the longitudinal design enabled us to investigate both pre-post treatment differences as well as the association between changes in interpersonal skills and metacognitive beliefs and depressive symptom severity. To our knowledge, this is the first study that investigated change in preoperational thinking and its association with symptom change in depression and that focused on change in both interpersonal as well as metacognitive skills over the course of treatment in depressed patients. Second, the observational design of our study increases the ecological validity of our findings (69). Thus, this increases the generalizability of our findings to real world clinical settings. Of note, sociodemographic data of this present naturalistic study are comparable to general population data (70).

There are also limitations that warrant discussion. First and foremost, we cannot establish temporal precedence of change in skills deficits before change in depressive symptoms. Due to the rather short treatment duration of 8 weeks, data regarding skills deficits was collected at baseline and end of treatment only in order to allow for treatment effects to take place. However, temporal precedence of the proposed mediating variable is often regarded a prerequisite when investigating mechanisms of change and establishing causal effects (71, 72). Nevertheless, the use of cross-sectional designs to test mediation is prevalent (72). The correlational evidence of this present study may be seen as an important starting point for future research (71, 73). Future studies should further investigate the proposed mechanisms of change by including additional data measurement points in order to allow for the investigation of mediation models by establishing temporal precedence. Due to the observational study design, patients were not randomly assigned to treatment with CBASP or MCT. Rather, choice of treatment was based on diagnosis, presenting complaints and patients' preferences. While we aimed to statistically control for observed baseline imbalances, we cannot account for unobserved confounding variables influencing our results. Future studies conducted as randomized controlled trials would minimize the influence of potential confounding effects (74). Due to the non-randomization, the treatment groups (CBASP vs. MCT) also differed in sample size. As unbalanced groups contribute to reduced power, future studies should pay attention to equally balanced groups in order to maximize power. Also due to the observational study design, sample size was determined by admission rate and capacities of the day clinic. With this present sample size, our study was only powered to detect large effects between treatments. This needs to be kept in mind when interpreting results (esp. concerning differential treatment effects) and future studies should focus on analyzing larger samples. As self-report questionnares were used, reporting biases may have influenced results. Also, as there was no separation of patient groups in common spaces of the day clinic, we could not control for eventual diffusion effects as patients may exchange information and experiences about their treatments. Also, as the majority of patients in this study suffered from PDD, generalizability to episodically depressive samples may be limited. Addressing this limitation, future studies could investigate whether diagnosis of depression may constitute a moderating factor to the association between improvement in interpersonal or metacognitive skills deficits and change in depressive symptoms. Finally, due to the small sample size at follow-up and thus low power to detect effects, long-term data should be further investigated as results may point toward friendly-dominant behavior change being associated with change in depressive symptomatology also at 10 months follow-up.

## Conclusion

We found that changes in interpersonal skills might be of relevance in reducing depressive symptomatology. Increases in friendly-dominant behaviors and a less preoperational style of thinking were associated with alleviation of depressive symptoms, thereby supporting McCullough's interpersonal model of depression. These findings also have implications for treatment as they emphasize the importance of addressing interpersonal challenges in the treatment of depression. Future research is needed to investigate potential moderators (e.g., chronicity of depression) and mediators of the association between change in interpersonal and metacognitive skills and change in depressive symptomatology.

## Data Availability Statement

The raw data supporting the conclusions of this article will be made available by the authors, without undue reservation.

## Ethics Statement

The studies involving human participants were reviewed and approved by Ethics Committee. The patients/participants provided their written informed consent to participate in this study.

## Author Contributions

JK with support from SG: conception and design. KO and SS: acquisition of data. SS and KO with support from JK: analysis of data. SS and JK: interpretation of data. SS: drafting of manuscript. All authors made substantial contribution to the manuscript and gave approval to the final version before submission.

## Conflict of Interest

JK received funding for clinical trials (German Federal Ministry of Health, Servier), payments for lectures on Internet interventions (Servier) and payment for workshops and books (Beltz, Elsevier, Hogrefe, Springer) on psychotherapy of chronic depression and psychiatric emergencies. EF received funding for a clinical trial comparing MCT and Behavioral Activation in the treatment of depression (Addisca gemeinnützige GmbH) and for a clinical trial in patients with Borderline Personality Disorder (Else Kröner-Fresenius Stiftung), payments for workshops, presentations and books (Beltz) on psychotherapy for depression and other disorders. The remaining authors declare that the research was conducted in the absence of any commercial or financial relationships that could be construed as a potential conflict of interest.

## Publisher's Note

All claims expressed in this article are solely those of the authors and do not necessarily represent those of their affiliated organizations, or those of the publisher, the editors and the reviewers. Any product that may be evaluated in this article, or claim that may be made by its manufacturer, is not guaranteed or endorsed by the publisher.
